# Evaluation of a Micronized Collagen Wound Matrix for Advanced Wound Management: Physical, Structural and Biochemical Properties

**DOI:** 10.1111/wrr.70186

**Published:** 2026-08-03

**Authors:** Rami A. Nasrallah, Jumana R. Alhamdi, Thuan‐Ethan Ngo, Justin T. Avery, Kelly A. Kimmerling, Katie C. Mowry

**Affiliations:** ^1^ Organogenesis Innovation Center San Diego California USA; ^2^ Organogenesis Discovery Center Birmingham Alabama USA

**Keywords:** acellular, biomaterial, collagen, matrix metalloproteinases, protease inhibition, wound management

## Abstract

Wounds affect millions worldwide and non‐healing wounds are characterised by excessive protease activity, impaired extracellular matrix remodelling and persistent inflammation, highlighting the need for innovative extracellular matrix‐based therapies. This study evaluated a micronized collagen wound matrix (Collagen Wound Matrix‐Micronized; CWM‐MZ), a biomaterial derived from porcine small intestinal submucosa and processed into a micronized form. Physical and structural properties of CWM‐MZ were characterised using collagen content and structure, sulphated glycosaminoglycan content, deoxyribonucleic acid removal, particle size and its ability to inhibit protease activity. Functional in vitro assays were used to assess biochemical responses using dermal fibroblasts and endothelial cells under conditions simulating wound fluid to evaluate effects on cell viability, migration, sulphated glycosaminoglycan production, collagen type I deposition and tube formation. Physically and structurally, CWM‐MZ preserved the native collagen architecture, was robustly decellularized and exhibited dose‐dependent inhibition of multiple proteases, achieving over 80% inhibition of matrix metalloproteinases. Using in vitro cell‐based assays, CWM‐MZ resulted in enhanced fibroblast viability, supported fibroblast migration, increased sulphated glycosaminoglycan and collagen type I production and led to more robust formation of capillary‐like structures by endothelial cells. These results highlight how CWM‐MZ may support an environment for wound healing and its continued evaluation as a novel extracellular matrix‐based biomaterial for wound management.

**Abbreviations**
BSA: bovine serum albumin
CWM‐MZ: Collagen Wound Matrix‐Micronized
ECM: extracellular matrix
EDTA: ethylenediaminetetraacetic acid
HUVEC: human umbilical vein endothelial cells
IF: immunofluorescence
LLSPSA: light scattering particle sizing analysis
MMP: matrix metalloproteinase
SEM: scanning electron microscopy
sGAG: sulphated glycosaminoglycans
SIS: small intestine submucosa
SWF: simulated wound fluid

## 1 Introduction

Wound healing is a complex biological process that restores tissue integrity following injury. Each year, millions of patients require wound care for complex acute wounds such as surgical dehiscence, traumatic injuries and surgical site infections. While most acute wounds progress through the normal stages of healing, complications can arise that delay recovery and increase healthcare costs. Chronic wounds, affecting around 40 million patients globally, represent a particularly challenging subset [1]. These wounds are characterised by prolonged healing times, resistance to conventional therapies and high recurrence rates. They pose a major clinical and economic burden, with estimated treatment costs exceeding $32 billion annually in the United States alone and over $100 billion worldwide [2]. Chronic wounds exhibit excessive protease activity, impaired extracellular matrix (ECM) remodelling and dysregulated inflammation, underscoring the need for innovative therapeutic approaches [3].

Understanding wound pathology necessitates understanding the skin's structure and function. The epidermis and dermis form a three‐dimensional network that provides mechanical support and biochemical cues essential for tissue repair. The epidermis acts as a barrier against environmental insults and is primarily comprised of keratinocytes supported by an underlying basement membrane [4]. Beneath the epidermis lies the dermis, a thicker, vascularized layer rich in ECM proteins such as collagen and elastin, which confer mechanical strength and elasticity to the skin [5]. Collagen, the most abundant protein in the dermis, plays a pivotal role in maintaining structural integrity. Dermal fibroblasts synthesise ECM components and secrete growth factors that support angiogenesis and keratinocyte function, while keratinocytes proliferate and migrate to cover the wound, ensuring the coordinated repair of the epidermal and dermal layers [6, 7]. Dysregulation of wound healing, particularly through the elevated activity of matrix metalloproteinases (MMPs), degrades essential ECM components in the dermis, perpetuates inflammation and impedes regeneration [8]. This imbalance significantly contributes to wound pathology, highlighting the importance of maintaining the structural and functional integrity of both the epidermis and dermis [9, 10].

Wound exudate is an essential component of wound healing and is primarily a leukocyte‐rich fluid secreted during the inflammatory phase of wound healing due to the vasodilation of blood vessels. Proper moisture balance is essential for healing and excessive exudate can cause complications, including skin maceration, increased infection risk, erosion of surrounding tissue and delayed healing [11]. Exudate from infected and chronic wounds stuck in the inflammatory phase has been associated with elevated levels of MMPs, which contribute to the breakdown of ECM and the stalling of the wound repair process [12]. The ideal dressing should maintain optimal moisture levels while remaining intact, resisting degradation and providing adequate absorption with good moisture retention.

Collagen dressings may undergo a range of modifications, including decellularization, preservation of the native structure versus hydrolysis, the addition of antimicrobials to manage bioburden, micronisation to increase surface area and improve contact with irregularly shaped wounds and cross‐linking to enhance durability and resist rapid degradation [13, 14]. For example, micronisation of collagen isolated from porcine urinary bladder matrix has found that proteolytic degradation of acellular ECM promoted cell‐specific recruitment and proliferation, which is critical for wound remodelling [15, 16]. Small intestine submucosa (SIS) stands out as a leading biomaterial candidate for tissue regeneration, with significant potential for repairing both soft and hard tissues. Its successful application in tissue engineering for organs such as the heart, bladder, skin and cartilage underscores its versatility and biocompatibility in the wound environment [17, 18, 19, 20].

Collagen Wound Matrix‐Micronized (CWM‐MZ) is an innovative ECM‐based biomaterial designed to address limitations of traditional wound care approaches. It is derived from porcine SIS and processed using chemical cleaning to decellularize and micronisation to particulate, resulting in preservation of the native three‐dimensional structure and bioactive components, including collagen. Collagen is widely reported in the literature to support cell attachment and extracellular matrix remodelling in vitro [17, 21, 22]. Removal of cellular components during processing reduces immunogenic material, while micronisation increases surface area, enabling increased absorption capacity and close contact with the wound bed. This manuscript investigates the potential of using CWM‐MZ for the management of wounds through a comprehensive in vitro analysis of its physical, structural and biochemical properties.

## 2 Methods

### 2.1 Scanning Electron Microscopy

A FEI Quanta 200 FEG MKII scanning electron microscope (SEM) (ThermoFisher Scientific, Waltham, MA) was used to examine the CWM‐MZ powder at UMass Chan Medical School (Worcester, MA). SEM imaging was captured at 1000×, 5000× and 40,000×, allowing for visualisation of D‐period banding of individual collagen fibrils. A minimum of three SEM images at 40,000× were obtained and 10 randomly selected D‐period bands were measured per image (*n* = 30 total D‐period bands). D‐periods were measured using ImageJ software (National Institute of Health, Bethesda, MD).

### 2.2 Particle Size Analysis

Particle size of a single lot of CWM‐MZ was analysed using laser light scattering particle sizing analysis (LLSPSA; Jordi Labs LLC, Mansfield, MA). The assay was performed on dry samples by adding 1/32 tsp. of CWM‐MZ to a turbotrac tray. Samples were analysed using a Microtrac S3500 tri‐laser system (Microtrac Retsch GmbH, Hann, Germany). Results are reported as a percentage of events by measured particle size.

### 2.3 Collagen Content

The total collagen content was measured using the Sircol Collagen Assay (Biocolor Life Science Ltd., Belfast, United Kingdom). Briefly, 25 mg of CWM‐MZ or unprocessed SIS tissue was mixed with 0.5 mL of cold acid‐pepsin solution and incubated at 4°C for 20 h to extract soluble collagen (*n* = 3 per group). The sample was then incubated at 65°C for 3 h with fragmentation reagent to recover insoluble collagen. Following incubation, the mixture was centrifuged at 10,000 × *g* for 10 min. The supernatant was collected and analysed for total collagen content using the Sircol assay, which specifically binds to collagen, using a SpectraMax M3 microplate reader (Molecular Devices LLC., San Jose, CA) set at 556 nm wavelength. Collagen content was normalised to the dry weight of the samples.

### 2.4 sGAG Content

The sulphated glycosaminoglycan (sGAG) content in CWM‐MZ and unprocessed SIS tissue was measured using the Blyscan Assay (Biocolor Life Science Ltd., Belfast, United Kingdom). For this assay, 20 mg of CWM‐MZ or unprocessed SIS tissue was incubated in 1 mL of papain extraction buffer at 65°C for 4 h (*n* = 3 per group). The papain extraction buffer consisted of 0.2 M sodium phosphate buffer (Na_2_HPO_4_–NaH_2_PO_4_, Sigma Aldrich Co., St. Louis, MO) at pH 6.4, supplemented with 8 mg/mL sodium acetate, 4 mg/mL ethylenediaminetetraacetic acid (EDTA, disodium salt), 0.8 mg/mL cysteine HCl and 0.1 mg/mL papain (all from Sigma Aldrich Co., St. Louis, MO). After incubation, the samples were centrifuged at 10,000 × *g* for 10 min. The supernatant was collected and analysed for sGAG using a SpectraMax M3 microplate reader set at 656 nm wavelength. sGAG content was normalised to the dry weight of the samples.

### 2.5 DNA Content

The DNA content in CWM‐MZ and unprocessed SIS tissue was determined using the GeneJET Genomic DNA Purification Kit (Invitrogen Co., Waltham, MA) following the manufacturer's instructions. For this analysis, approximately 16 mg of hydrated CWM‐MZ or unprocessed SIS tissue was used (*n* = 3 per group). DNA concentration was measured with a NanoDrop ND‐1000 (NanoDrop Technologies Inc., Wilmington, DE) and quantified on a SpectraMax M3 microplate reader at 260 and 280 nm wavelengths. DNA content was normalised to the dry weight of the samples.

### 2.6 Protease Inhibition Assays

Protease inhibition by CWM‐MZ was evaluated using fluorescent substrate–based assays following manufacturer protocols. For all assays, CWM‐MZ was tested at concentrations of 25, 10 or 5 mg/mL. Each assay was performed in three independent runs, with three replicates per condition per run (*n* = 9 total per CWM‐MZ concentration). Samples at each CWM‐MZ concentration were hydrated in deionised water, sonicated with a direct probe for 30‐s intervals over four cycles, incubated in a sonication bath for 45 min and then centrifuged at 10,000 × *g* for 10 min. The resulting supernatants were collected for analysis.

To assess total protease inhibition, the CWM‐MZ supernatant was mixed with trypsin at a final concentration of 300 ng/mL for 30 min at room temperature and activity was measured using the Pierce Fluorescent Protease Assay Kit (ThermoFisher Scientific, Waltham, MA). Fluorescence readings of CWM‐MZ supernatant with 300 ng/mL trypsin were background‐corrected by subtracting CWM‐MZ supernatant alone and compared to the trypsin control at 300 ng/mL. Fluorescence intensity was measured using a SpectraMax M3 microplate reader at excitation/emission of 485/538 nm.

For elastase inhibition, the EnzChek Elastase Assay Kit (ThermoFisher Scientific, Waltham, MA) was used with porcine pancreatic elastase at a final concentration of 10 U/mL for 30 min at room temperature. The substrate, DQ elastin (BODIPY FL‐labelled), is quenched until digested by elastase. Fluorescence readings of CWM‐MZ supernatant with elastase (10 U/mL) were background‐corrected by subtracting CWM‐MZ supernatant alone and compared to the elastase control at 10 U/mL. Measurements were collected on a SpectraMax M3 at excitation/emission 505/515 nm.

For gelatinase and collagenase inhibition assay, the EnzChek Gelatinase/Collagenase Assay Kit (ThermoFisher Scientific, Waltham, MA) was used with Clostridium collagenase (0.2 U/mL final concentration) for 30 min at room temperature. The substrate, DQ gelatin (fluorescein‐labelled), produces fluorescence upon enzymatic digestion. Fluorescence readings of CWM‐MZ supernatant with collagenase were background‐corrected by subtracting CWM‐MZ supernatant alone and compared to the collagenase control (0.2 U/mL final concentration). Data were collected on a SpectraMax M3 microplate reader at excitation/emission wavelengths of 495/515 nm.

### 2.7 MMP Inhibition Assay

To evaluate the inhibitory effects of CWM‐MZ on a panel of the most common MMPs, an MMP inhibition assay was conducted using Fluorometric Drug Discovery Kits (Enzo Life Sciences, Farmingdale, NY) according to the manufacturer's protocol. A sample of 6.0 mg of CWM‐MZ was hydrated in PBS for 1 h at room temperature in round‐bottom 96‐well plates (*n* = 3 per MMP assessed). After hydration, the sample was centrifuged, PBS was removed and MMP Assay Buffer was added to each well containing CWM‐MZ. Non‐inhibited MMP positive controls and a broad‐spectrum MMP inhibitor (NNGH inhibitor), both provided with the kit, were run alongside test samples. The plates were incubated at 37°C for 1 h to allow for inhibition. Following incubation, the MMP Assay Buffer exposed to the product was transferred to wells in a half‐volume 96‐well white NBS microplate (Millipore Sigma, Burlington, MA). The plates were then placed into a plate reader (Synergy H1, BioTek, Winooski, VT) set to excitation/emission wavelengths of 328/420 nm. Readings were taken every minute for 10 min. Linear regression was performed for each sample and the slope was used to calculate the percentage of MMP remaining using the following formula:

$$\text{Percent}\;\mathrm{MMP}\;\text{remaining}=\left(\frac{\text{Slope Unknown}}{\text{Slope Control}}\right)\times 100$$

Here, ‘Slope Unknown’ refers to the slope of a test article and ‘Slope Control’ refers to the average slope of the positive control wells. To determine the percentage of MMP inhibited, the percentage of remaining MMP was subtracted from 100%.

### 2.8 Assessment of Fibroblast Viability and Function in Simulated Wound Environment

To assess the effects of CWM‐MZ on cell growth under simulated wound conditions, a commercially available transwell insert system (Corning Inc., Corning, NY) was used to separate CWM‐MZ from the cells seeded below. The permeable membrane (8 μm pore size) was deemed suitable to allow for the diffusion of key molecules. Simulated wound fluid (SWF) is a buffer formulation that is designed to mimic the protein content, ionic strength and buffering capacity of clinical wound exudate. The SWF solution contained 5.844 g sodium chloride (NaCl), 3.3604 g sodium hydrogen carbonate (NaHCO_3_), 0.2982 g potassium chloride (KCl), 0.2775 g calcium chloride (CaCl_2_) and 33.0 g bovine albumin per litre of deionised water. Where indicated, collagenase type II (Worthington Biochemical Co., Lakewood, NJ) was added at specified U/mL to simulate protease‐rich chronic wound fluid [17]. Human dermal neonatal fibroblasts were isolated as described previously [23] under full informed consent. The fibroblasts maintained in DMEM with 10% FBS at passage 6 were seeded into 24‐well plates at a density of 4500 cells/cm^2^. Transwell inserts containing SWF with or without CWM‐MZ (8 mg/cm^2^) were placed on top of the wells containing cultured cells in media. The cells were incubated under physiologically relevant conditions (5% CO_2_, 37°C) for 2, 5 and 7 days. Cell viability was then assessed using CellTiter‐Glo 3D Cell Viability Assay (Promega Co., Madison, WI). Cells at known densities were used to establish a standard curve for quantitative analysis. Human dermal fibroblasts were counted using a NucleoCounter NC‐200 and serially diluted in media to obtain a range of defined cell numbers. Each dilution was prepared in duplicate and processed in parallel with experimental samples. CellTiter‐Glo reagent was added to each sample and standard according to the manufacturer's protocol and incubated for 10 min at room temperature before being analysed on a SpectraMax M3 microplate reader. Experiments were performed using three independent runs with *n* = 4 replicates per group.

Furthermore, to evaluate the effects of CWM‐MZ on cellular production of sGAG, human dermal fibroblasts were seeded into 24‐well plates at a density of 10,000 cells/cm^2^. Transwell inserts containing 0.5 mL of SWF with or without CWM‐MZ (8 mg/cm^2^) were placed on top of the wells containing cells in 1.5 mL low serum media (*n* = 3 per condition). The transwell inserts and cells were incubated under physiologically relevant conditions (5% CO_2_, 37°C) for 3, 5 and 7 days. Spent media were collected on Days 3, 5 and 7 and assessed for sGAG content using the Blyscan Assay (Biocolor Life Science Ltd., Belfast, United Kingdom) following the manufacturer's instructions using a SpectraMax M3 microplate reader set at 656 nm wavelength. Experiments were performed using three independent runs with *n* = 3 replicates per group.

To evaluate the inhibitory effects of CWM‐MZ on proteases within this environment, human dermal fibroblasts were seeded into 24‐well plates at a density of 10,000 cells/cm^2^. Transwell inserts containing 0.5 mL of SWF with collagenase type II (SWF+) at concentrations of 13 CDU/mL or 26 CDU/mL, with or without CWM‐MZ (8 mg/cm^2^), were placed on top of the wells containing cells in 1.5 mL of low serum (2% FBS) media. Additionally, wells containing fibroblasts maintained in standard growth media without any exposure to insults were included as a control for normalisation, which represented 100% viability. The transwell inserts and cells were incubated under physiologically relevant conditions (5% CO_2_, 37°C) for 4 h. Cell viability was then assessed using the CellTiter‐Glo Luminescent Cell Viability Assay (Promega Co., Madison, WI). Experiments were performed using three independent runs with *n* = 3 replicates per group.

### 2.9 Wound Scratch Assay

To investigate the effects of CWM‐MZ on fibroblast migration, a wound scratch assay was performed using conditioned media generated from transwell inserts. Transwell inserts containing 0.5 mL of SWF with or without CWM‐MZ (8 mg/cm^2^) were supplemented with collagenase type II to a final concentration of 20 CDU/mL and placed above 1.5 mL of low‐serum media (2% FBS) in the lower compartment. Inserts were incubated for 3 days at 37°C with 5% CO_2_ to allow equilibration. The resulting conditioned media was collected and applied to wounded/scratched fibroblast monolayers cultured in low‐serum conditions.

Primary human dermal fibroblasts were seeded at 9000 cells/cm^2^ in rectangular cell culture plates (EMD Millipore Corp., Burlington, MA) and cultured in full serum media for 72 h. Cells were then transitioned to low‐serum media to inhibit substantial proliferation [24, 25]. To confirm that wound closure was due to migration and not proliferation, select experiments included pretreatment with mitomycin C (10 μg/mL, 2 h at 37°C) in all experimental groups. Once confluent, monolayers were established, the media was aspirated and a cell comb (EMD Millipore Corp., Burlington, MA) was used to generate multiple horizontal scratches. Cells were washed with Dulbecco's PBS (DPBS) without Ca^2+^/Mg^2+^ and then treated with conditioned media from transwell inserts (SWF ± CWM‐MZ ± collagenase type II). Four treatment groups were tested: (1) SWF only (control), (2) SWF + CWM‐MZ, (3) SWF + collagenase type II and (4) SWF + collagenase type II + CWM‐MZ. Wound scratch assays were performed using three independent runs with *n* = 5 replicates per group, with an additional confirmatory experiment performed using mitomycin C. Cells were incubated under physiologically relevant conditions (5% CO_2_, 37°C) and images were captured at 20× magnification (Olympus DP75, CellSens v.4.2.1, Evident Scientific Inc., Waltham, MA) for up to 66 h post‐scratch. Within each plate, five scratch sites were selected for analysis. At each site, the gap distance was measured at four equidistant points and the average gap closure was calculated relative to 0 h. Positions where opposing cell fronts had fully merged were considered 100% closed (gap distance = 0), whereas any remaining acellular area between fronts was scored as incomplete closure. Percent closure per scratch was calculated as the mean across these positions.

### 2.10 Tube Formation Assay

To evaluate the angiogenic responses of Human Umbilical Vein Endothelial Cells (HUVECs, ThermoFisher Scientific, C0035C, Lot 2666768) in response to a simulated wound environment with CWM‐MZ, a tube formation assay was conducted using conditioned media generated from transwell inserts (as described above) containing SWF ± CWM‐MZ (8 mg/cm^2^). The resulting conditioned media were collected and applied to 96‐well plates with HUVECs as described below.

Tube formation assays were performed using Cultrex gelled basement membrane extract (Cultrex, R&D Systems, Minneapolis, MN), with HUVECs seeded at a density of 7.8 × 10^4^ cells/cm^2^ on a 96‐well plate on the gelled basement membrane and incubated with conditioned media for 12 h. After the incubation period, cells were stained with Calcein‐AM (R&D Systems, Minneapolis, MN) to visualise capillary‐like structures. Sulforaphane (10 μM) was incorporated into standard endothelial cell culture media as an inhibitor of tube formation (negative control), according to the R&D Systems Tube Formation Assay Kit protocol (Cat. 3470‐096‐K, R&D Systems, Minneapolis, MN). The experiment was repeated in three independent runs with 4 replicates per group. Quantitative analysis was conducted using ImageJ software. For tube length measurements, using the straight‐line tool in the ImageJ software, a line was manually drawn to measure each branch within each image, using the ROI measured using the straight‐line tool in ImageJ and calibrated to scale. For the number of nodes, a multipoint tool in ImageJ software was used to manually count the number of nodes in each image.

### 2.11 ECM Deposition and Cytoskeletal Protein Expression

To evaluate ECM deposition and cytoskeletal remodelling in response to CWM‐MZ, primary human dermal fibroblasts were seeded at 1500 cells/cm^2^ in 12‐well plates and cultured in fibroblast growth media (10% FBS) under physiologically relevant conditions (5% CO_2_, 37°C) until 100% confluent. Transwell inserts containing SWF with or without CWM‐MZ (8 mg/cm^2^; *n* = 3 wells per group) were placed into wells containing low serum (2% FBS) growth media in the lower compartment. Cultures were maintained for up to 21 days. Only the lower‐compartment media was refreshed every 2–3 days; the SWF ± CWM‐MZ solution in the transwell inserts (upper compartment) was left undisturbed to allow continuous generation of conditioned media. On Days 4, 14 and 21, cells were fixed with 4% paraformaldehyde, permeabilized with 0.1% Triton X‐100 and blocked with 2% BSA before immunostaining, as summarised in Table 1.

**TABLE 1:** Antibodies and stains used for immunofluorescence.

Target	Reagent/catalogue no.	Dilution	Incubation conditions	Secondary antibody/readout
Collagen I	Polyclonal Ab (PA126204, Thermo Fisher)	1:200	Overnight, 4°C, shaker 115 rpm	Alexa Fluor 568 Goat anti‐Rabbit IgG (A11011, Thermo Fisher)
Vimentin	Monoclonal Ab (OMA1‐06001, Thermo Fisher)	1:200	Overnight, 4°C, shaker 115 rpm	Alexa Fluor 488 Donkey anti‐Mouse IgG (A21202, Thermo Fisher)
F‐Actin	ActinRed 555 ReadyProbes (R37112, Thermo Fisher)	Ready‐to‐use	30 min, RT	TRITC channel
Nuclei	NucBlue Fixed Cell ReadyProbes (DAPI), R37606, Thermo Fisher	Ready‐to‐use	30 min, RT	DAPI channel

Fluorescence images were acquired at 40× magnification using an Olympus DP75 digital camera mounted on an IX83 inverted microscope (Olympus Corp., Tokyo, Japan) and analysed with CellSens Dimension v.4.2.1 (Evident Scientific Inc., Waltham, MA) and ImageJ v.1.54 k. A minimum of three images per well were collected and representative images are shown. Manufacturer protocols (ThermoFisher Scientific, Waltham, MA) were followed for antibody and staining procedures.

### 2.12 Statistical Analyses

Means, medians and standard deviations were calculated for each assay. Statistical analyses were performed using GraphPad Prism software (GraphPad Software, San Diego, CA). Depending on the experimental design, comparisons between the CWM‐MZ and control groups were conducted using either unpaired Student's *t*‐tests, two‐way repeated measures analysis of variance (ANOVA) with post hoc Šídák's multiple comparisons test or two‐way ANOVA with post hoc Tukey multiple comparisons test. Significance was denoted as *p < 0.05.

## 3 Results

### 3.1 CWM‐MZ Consists of Native, Micronized Collagen

CWM‐MZ has an off‐white appearance and consists of fine, opaque white particulates, resulting in a powder that can be dispersed over a large surface area and/or fit into irregularly shaped or tunnelled wounds (Figure 1A). The surface morphology and microstructure of CWM‐MZ were assessed using SEM, with images of CWM‐MZ taken at 40,000× magnification, allowing for D‐period banding of individual collagen fibrils to be observed (Figure 1B). The average D‐period length was 66.43 ± 6.40 nm (*n* = 30). SEM images depict a micronized collagen network with a high degree of porosity (Figure 1B). Particle size was measured using laser light scattering particle sizing analysis (LLSPSA); the average particle size of PPMZ was 334 μm, with a range between 28.53 and 1184 μm (Figure 1C). Biochemical analysis revealed that CWM‐MZ exhibited a collagen composition comparable to unprocessed tissue measured by Sircol assay (Figure 1D). A significant reduction in sulphated glycosaminoglycan (sGAG) content was observed in CWM‐MZ compared to unprocessed tissue, indicating that the processing effectively removed the majority of sGAG content (Figure 1E). Similarly, DNA was at undetectable levels in CWM‐MZ compared to unprocessed tissue, indicating successful, robust decellularization of CWM‐MZ (Figure 1F).

**FIGURE 1:**
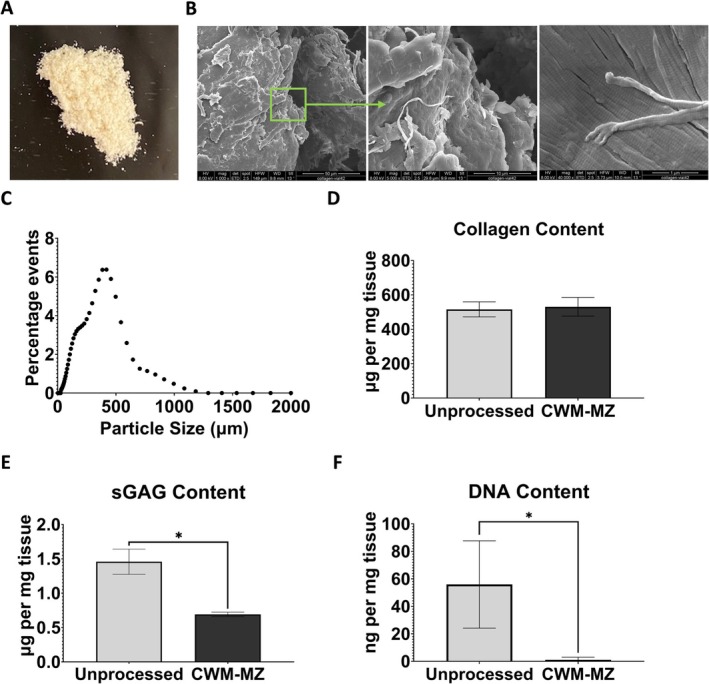
Physical, structural and biochemical characterisation of Collagen Wound Matrix‐Micronized (CWM‐MZ). (A) Macroscopic appearance of CWM‐MZ as a dry, fine powder. (B) Scanning electron microscopy (SEM) images showing preserved native collagen fibril architecture and characteristic D‐period banding. Images captured at 1000×, 5000× and 40,000× magnification; scale bars = 50, 10 and 1 μm, respectively. (C) Particle size distribution of a single lot of dry CWM‐MZ measured by laser light scattering particle sizing analysis (LLSPSA). (D) Total collagen content measured by Sirius Red assay in unprocessed dry tissue and CWM‐MZ. (E) Sulphated glycosaminoglycan (sGAG) content in unprocessed wet tissue and CWM‐MZ. (F) DNA content in unprocessed wet tissue and CWM‐MZ. *N* = 3 for each group in all assays. Data are shown as mean ± standard deviation; *p < 0.05 from unpaired Student's *t*‐test.

### 3.2 CWM‐MZ Inhibits Proteolytic Activity

With elevated proteolytic activity being a hallmark of chronic wound pathology, protease and MMP inhibition assays were performed to determine the properties of CWM‐MZ in the context of these environmental insults. Exposure to increasing concentrations of CWM‐MZ resulted in progressively greater inhibition of multiple proteases (Figure 2A–D). When assessed against trypsin, CWM‐MZ reduced activity by 66%, 45% and 24% at 25, 10 and 5 mg/mL, respectively, with statistically greater inhibition observed with increasing concentration of CWM‐MZ (Figure 2A). Collagenase and gelatinase activities were similarly inhibited in a dose‐dependent manner, with reductions of 79%, 66% and 46% at the same concentrations, again showing statistically greater inhibition with increasing concentration (Figure 2B). Elastase activity was also inhibited, with 80%, 70% and 43% reduction at 25, 10 and 5 mg/mL, respectively; however, statistical differences between 10 and 25 mg/mL were not observed (Figure 2C).

**FIGURE 2:**
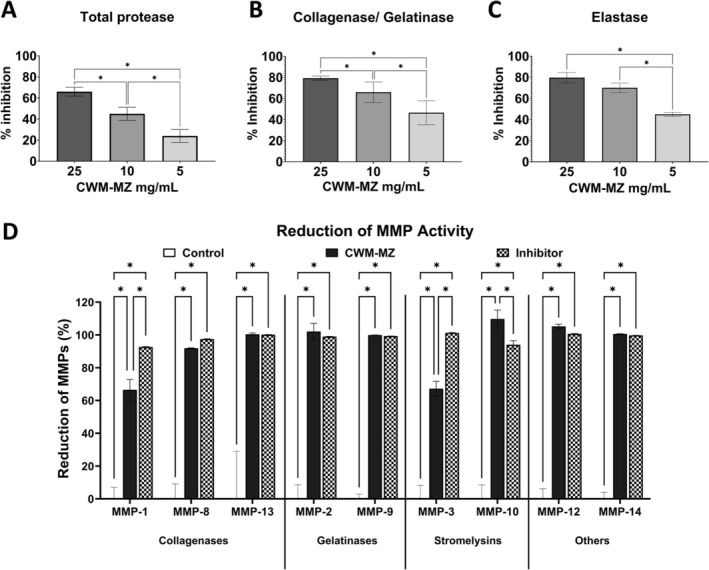
Dose‐dependent inhibition of protease activity by Collagen Wound Matrix‐Micronized (CWM‐MZ). (A) Total protease inhibition (*n* = 9 per concentration), (B) collagenase/gelatinase inhibition (*n* = 9 per concentration) and (C) elastase inhibition at CWM‐MZ concentrations of 25, 10 or 5 mg/mL (*n* = 6 per concentration). (D) Inhibition of individual matrix metalloproteinases (MMPs). *N* = 3 per MMP tested. Data are presented as mean ± standard deviation. *p < 0.05 from two‐way ANOVA.

To further characterise these effects, inhibition of individual matrix metalloproteinases (MMPs) was evaluated (Figure 2D). CWM‐MZ demonstrated broad‐spectrum activity across multiple MMP subtypes. Among the collagenases, MMP‐1 activity was reduced by 65%–70%, while MMP‐8 and MMP‐13 were inhibited by 90%. Both gelatinases, MMP‐2 and MMP‐9, showed strong suppression, with inhibition exceeding 85%. Stromelysins displayed variable responses, with MMP‐3 inhibited by 60% and MMP‐10 nearly completely inhibited (> 95%). Other MMPs, including MMP‐12 and MMP‐14, were strongly inhibited, with activity reduced by greater than 90%. In all assessments, statistical inhibition was observed compared to control; however, statistical differences between the broad‐spectrum MMP inhibitor NNGH and CWM‐MZ were observed for MMP‐1, MMP‐3 and MMP‐10.

### 3.3 CWM‐MZ Supports Fibroblast Growth, sGAG Production and Preserves Cell Viability In Vitro

To evaluate the effects of CWM‐MZ under proteolytic stress, human dermal fibroblasts were cultured in SWF ± CWM‐MZ. The in vitro transwell model provided a defined microenvironment that allows precise control over factors influencing cell behaviour, including chemical gradients and extracellular matrix (ECM) components, while minimising obstruction from CWM‐MZ particulates during image analysis and observations in vitro (Figure 3A).

**FIGURE 3:**
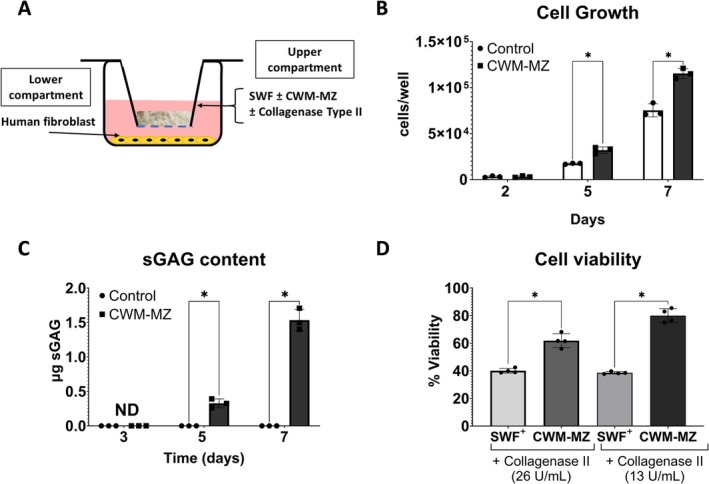
Effects of Collagen Wound Matrix‐Micronized (CWM‐MZ) on fibroblast growth, protease resistance and extracellular matrix production in a simulated wound model. (A) Schematic of the transwell system simulating chronic wound conditions. (B) Cell growth in simulated wound fluid (SWF) ± CWM‐MZ. (C) Sulphated glycosaminoglycans (sGAG) content in spent media following exposure to SWF ± CWM‐MZ. (D) Cell viability under proteolytic stress [SWF + collagenase II (SWF+)] ± CWM‐MZ. 3 independent runs, 3 replicates per experiment per group. Data are shown as mean ± standard deviation; *p < 0.05 from two‐way repeated measures ANOVA. U, units.

Normal human dermal fibroblasts cultured with simulated wound fluid (SWF) and CWM‐MZ demonstrated a significant increase in cell growth compared to cells cultured with SWF alone. Fibroblast cell density showed no differences between groups at Day 2, but by Day 5, cultures treated with CWM‐MZ contained significantly greater cell density than SWF controls (31,967 vs. 17,300 cells/well; *p* = 0.049). This effect was further amplified by Day 7, where CWM‐MZ cultures reached 115,333 cells/well compared with 75,300 cells/well in controls (*p* = 0.0058) (Figure 3B).

Analysis of spent media revealed that fibroblasts cultured with CWM‐MZ secreted significantly higher levels of sulphated glycosaminoglycans (sGAGs) compared with SWF controls. By Days 5 and 7, sGAG production in the CWM‐MZ supplemented group reached 0.4 and 1.6 μg per well, respectively, whereas SWF alone remained non‐detectable (ND) at both timepoints (*p* < 0.001; Figure 3C).

To evaluate fibroblast viability under high protease conditions mimicking a chronic wound environment, cells were cultured in simulated wound fluid (SWF) ± CWM‐MZ with exposure to two concentrations of collagenase type II (26U and 13U). Viability was benchmarked against fibroblasts maintained in standard growth media without any insults, which represented 100% viability. At 26U of collagenase, fibroblasts cultured in SWF alone had a mean viability of 40.1%, whereas the addition of CWM‐MZ resulted in a viability of 61.9% (Figure 3D, *p* = 0.0006). At a lower level of collagenase, SWF alone reduced viability to 38.6%, while the presence of CWM‐MZ further improved viability to 80.1% (*p* < 0.0001).

### 3.4 CWM‐MZ Supports Closure in an Elevated Proteolytic Environment In Vitro

The rate of wound closure by human dermal fibroblasts cultured in media conditioned with either SWF ± CWM‐MZ in the presence or absence of collagenase type II was assessed. Wound scratches were created on a confluent layer of human dermal fibroblasts and then treated with conditioned media as described above (Figure 4A). For all groups, changes were most evident after 24 h, but all time points are reported in Figure 4B. The SWF control group showed progressive closure over time. CWM‐MZ significantly enhanced gap closure compared to the SWF control at 42 and 48 h (Figure 4B). By 66 h, wounds appeared largely closed in all groups; however, small residual non‐confluent regions remained more frequently in SWF‐treated cultures, yielding a modest but measurable difference in percent closure based on our gap distance scoring (Figure 4B). The addition of collagenase II to the model impaired closure, with mean values of 33% at 42 h, 39% at 48 h and 53% at 66 h. When CWM‐MZ was included in the SWF + collagenase II model, closure rates were partially rescued to 48% at 42 h, 55% at 48 h and 76% at 66 h (Figure 4B). To confirm that observed closure reflected migration rather than proliferation, one additional experiment included pretreatment with mitomycin C. Under these conditions, cells treated with SWF + CWM‐MZ still showed enhanced closure compared to SWF alone, supporting a migration‐dependent effect (96.9% vs. 65.6% closure at 66 h).

**FIGURE 4:**
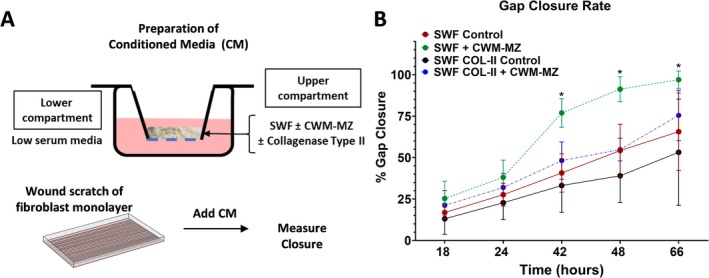
Collagen Wound Matrix‐Micronized (CWM‐MZ) enhances dermal fibroblast migration and gap closure under proteolytic conditions. (A) Schematic of the wound scratch assay using conditioned media. (B) Quantification of gap closure analysis over time with simulated wound fluid (SWF) ± CWM‐MZ (conditioned media) ± Collagenase (COL) II. 3 independent runs, 5 replicates per experiment per group. Data are presented as mean ± standard deviation; *p < 0.05 from two‐way repeated measures ANOVA.

### 3.5 CWM‐MZ Enhances Tube Formation in Human Umbilical Vein Endothelial Cells In Vitro

The ECM provides both structural support and bioactive properties to support endothelial cells in forming stable and organised tube‐like structures. A tube formation assay using human umbilical vein endothelial cells (HUVECs) was used to better understand how conditioned media generated from CWM‐MZ might impact these processes in vitro. Three groups were assessed: a negative control containing Sulforaphane (10 μM; known inhibitor of tube formation), SWF alone (control group) and SWF with CWM‐MZ. As expected, the Sulforaphane negative control minimised tube formation, confirming assay sensitivity. In the SWF + CWM‐MZ group, there was a significant increase in the overall qualitative length and complexity of tubes formed by HUVEC cells compared to SWF alone (Figure 5A). HUVEC cells cultured in the presence of CWM‐MZ conditioned media resulted in an increase in the number of nodes (Figure 5B), with 74.9 nodes compared to 39.4 nodes in the control group. There was a corresponding significant increase in the length of capillary‐like structures in HUVEC cells following treatment with SWF + CWM‐MZ conditioned media compared to control (*p* = 0.0069; Figure 5C).

**FIGURE 5:**
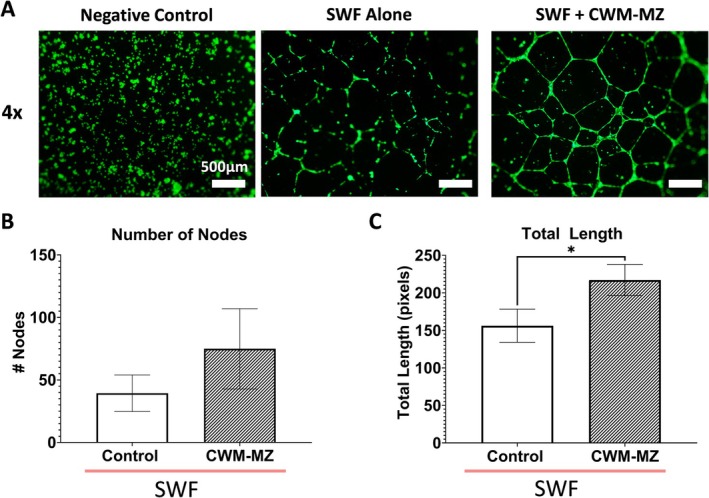
Collagen Wound Matrix‐Micronized (CWM‐MZ) promotes endothelial cell angiogenic activity in vitro. (A) Representative images of human umbilical vein endothelial cells (HUVECs) stained with Calcein‐AM after 12 h in conditioned media (4× objective). (B) Quantification of total node counts. (C) Total tube length measured using ImageJ software. 3 independent runs, 4 replicates per experiment per group. Data are shown as mean ± standard deviation; *p < 0.05 from unpaired Student's *t*‐test. Scale bar = 500 μm.

### 3.6 CWM‐MZ Supports Human Fibroblast ECM Deposition In Vitro

Immunofluorescence staining was performed to evaluate the deposition of collagen type I as well as the expression of vimentin and filamentous actin (F‐actin) in confluent human fibroblast cultures. Using a transwell model, cells were cultured with SWF ± CWM‐MZ for 4, 7 or 21 days (Figure 6).

**FIGURE 6:**
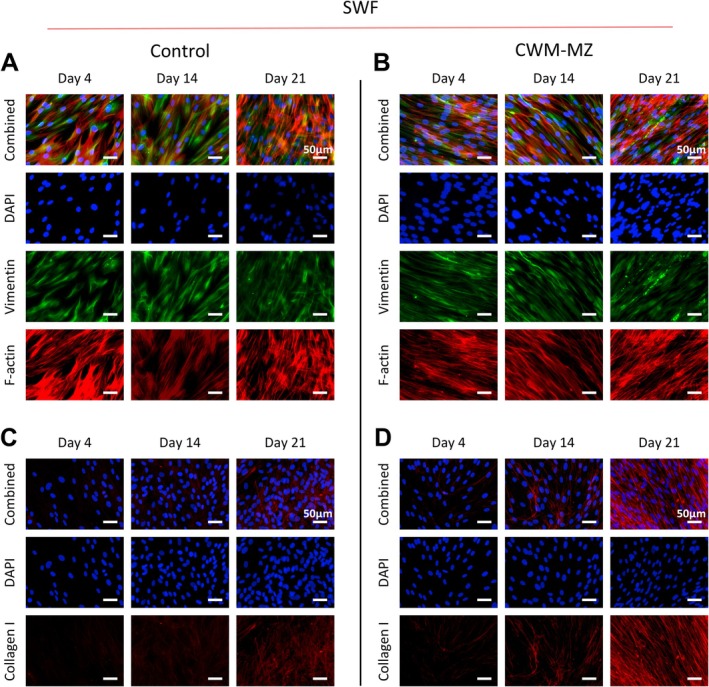
Immunofluorescent staining of dermal fibroblasts cultured with simulated wound fluid (SWF) ± Collagen Wound Matrix‐Micronized (CWM‐MZ) in a transwell model. Representative images at Days 4, 14 and 21 showing (A, B) vimentin (green) and F‐Actin (red) staining or (C, D) Collagen I (red) staining. Nuclei were stained with DAPI (blue). Images acquired at 40× magnification. Scale bar = 50 μm. DAPI, 4′,6‐diamidino‐2‐phenylindole.

Fibroblast cells maintained in media with SWF alone exhibited less ECM deposition, with weaker staining for collagen type I and vimentin compared to cultures containing CWM‐MZ. In contrast, fibroblasts cultured with SWF + CWM‐MZ demonstrated more robust ECM protein deposition, characterised by stronger and more organised collagen type I (red) and expression of vimentin (green) and F‐actin (red) staining, along with larger numbers of nuclei (DAPI, blue). These differences became most evident by Day 21, when ECM deposition in the SWF + CWM‐MZ group was markedly greater than in the SWF alone group.

## 4 Discussion

The use of CWM‐MZ within our in vitro models revealed findings relevant to its biochemical, structural and functional properties. CWM‐MZ retained key ECM components and effectively inhibited an array of proteases and MMPs. In fibroblast culture models, inclusion of CWM‐MZ was associated with improved cell viability, along with increased cell density and migration. Additionally, we found improved tube formation in a HUVEC model and more robust expression of vimentin and F‐actin along with deposition of collagen I in a fibroblast model. Overall, these results contribute to an understanding of how ECM‐based biomaterials support key processes important to tissue repair in vitro.

Environmental factors, such as ongoing elevated MMPs and elastase levels in chronic wound beds, disrupt collagen formation and hinder the wound healing cascade [12]. MMPs are critically important in normal wound healing, where transient increases facilitate ECM remodelling and cell migration. However, chronic wounds are characterised by persistently elevated and dysregulated MMP expression, which degrades ECM at a faster rate than it can be deposited and disrupts angiogenesis [26, 27, 28, 29]. CWM‐MZ effectively inhibited a wide range of proteases, including trypsin, elastase, collagenases, gelatinases, stromelysins and other MMPs. The near‐complete suppression of multiple clinically relevant MMPs underscores its broad‐spectrum protease inhibitory activity in environments mimicking chronic wounds. By regulating protease activity [30], SIS‐based biomaterials such as CWM‐MZ may support preservation of the structural integrity of the wound environment.

Delayed fibroblast recruitment and impaired ECM synthesis are hallmark features of chronic wounds, where elevated protease activity and persistent inflammation disrupt collagen deposition and cell migration [31]. Dressings with biocompatible materials (e.g., collagen, hyaluronic acid) can support cell adhesion, migration and proliferation [32]. In this context, the ability of CWM‐MZ to preserve fibroblast viability in vitro aligns with previous reports showing that ECM‐derived scaffolds can stabilise the wound microenvironment and restore fibroblast function [33, 34]. Furthermore, acellular collagen dermal matrices have been shown to allow for the migration and infiltration of fibroblasts, making topical collagen dressings effective at promoting healing of diabetic wounds [31]. Enhanced scratch closure observed with CWM‐MZ‐conditioned media is consistent with studies demonstrating that protease‐modulating biomaterials promote more efficient fibroblast migration and contractile force generation required for wound closure [35, 36].

Profound effects were seen in the expression and deposition of ECM molecules with CWM‐MZ present in human dermal fibroblast culture with SWF. Fibroblasts exposed to CWM‐MZ secreted higher levels of sGAGs compared to SWF alone. Increased sGAG production in vivo is thought to signify a transition from the inflammatory to the proliferative phase of wound healing [33], potentially by direct binding to inflammatory cytokines and reducing excessive inflammation [34]. Collagen type I, the primary structural protein in the dermis, plays a key role in providing tensile strength and maintaining skin integrity [37, 38]. During the proliferative phase of healing, fibroblasts migrate into the wound bed, differentiate into myofibroblasts and begin synthesising ECM proteins. Elevated levels of F‐actin and vimentin highlight the activated status of the fibroblasts and have been shown to be important in both promoting cell migration as well as wound closure [39, 40, 41]. This was demonstrated herein with increased closure rates in the scratch assay and subsequent collagen deposition in the IF images. Initially, collagen type III is deposited, but as healing progresses, it is replaced by collagen type I to restore the tensile properties of the dermis [42]. Over 21 days of culture, fibroblasts with CWM‐MZ exhibited robust and increasing collagen type I deposition over time. Characterisation of these matrices in the context of fibroblast responses in vitro helps to contribute to the overall understanding of how to best utilise CWM‐MZ in wound management.

There are limitations in the current study including studies performed were in vitro, using a subset of cell types lacking biodiversity found within an in vivo wound bed. Expanding studies into the inclusion of additional cell types, like diabetic fibroblasts, keratinocytes and immune cells, would contribute to further understanding the wound healing process. Furthermore, due to the in vitro nature of these studies, complications to ECM remodelling and repair, such as fibrosis, could not be studied. Therefore, additional in vivo studies would further support understanding around the complex nature of wound healing and the interaction of collagen dressings with the wound environment.

The current work assessed the physical, structural and biochemical characteristics of a native collagen ECM matrix of CWM‐MZ as well as in vitro models elucidating its impact on a range of cell types. CWM‐MZ supports cell adhesion, migration and proliferation, along with protease inhibition in vitro. Future in vitro and in vivo studies are warranted to fully explore its impact on advanced wound care management.

## Author Contributions

R.A.N. contributed to study conceptualization and design; performed and supervised experimental work; conducted data analysis and interpretation; prepared figures; drafted the manuscript and contributed to critical revisions. J.R.A. contributed to experimental execution, data acquisition and data analysis; participated in manuscript revision. T.‐E.N. contributed to experimental execution, data acquisition and data analysis; participated in manuscript revision. J.T.A. contributed to experimental execution, data acquisition and data analysis; participated in manuscript revision. K.A.K. contributed to study design, interpretation of data and critical revision of the manuscript. K.C.M. provided overall project supervision; contributed to study conceptualization, experimental design oversight, interpretation of data and critical manuscript revision. All authors reviewed and approved the final manuscript and agreed to be accountable for all aspects of the work.

## Funding

This work was supported by Organogenesis.

## Conflicts of Interest

Rami A. Nasrallah, Jumana R. Alhamdi, Thuan‐Ethan Ngo, Justin T. Avery, Kelly A. Kimmerling and Katie C. Mowry are employees of Organogenesis.

## Data Availability

The data that support the findings of this study are available on request from the corresponding author. The data are not publicly available due to privacy or ethical restrictions.
